# The Emergence of Somatotopic Maps of the Body in S1 in Rats: The Correspondence Between Functional and Anatomical Organization

**DOI:** 10.1371/journal.pone.0032322

**Published:** 2012-02-29

**Authors:** Adele M. H. Seelke, James C. Dooley, Leah A. Krubitzer

**Affiliations:** 1 Center for Neuroscience, University of California Davis, Davis, California, United States of America; 2 Department of Psychology, University of California Davis, Davis, California, United States of America; University of New South Wales, Australia

## Abstract

Most of what we know about cortical map development and plasticity comes from studies in mice and rats, and for the somatosensory cortex, almost exclusively from the whisker-dominated posteromedial barrel fields. Whiskers are the main effector organs of mice and rats, and their representation in cortex and subcortical pathways is a highly derived feature of murine rodents. This specialized anatomical organization may therefore not be representative of somatosensory cortex in general, especially for species that utilize other body parts as their main effector organs, like the hands of primates. For these reasons, we examined the emergence of whole body maps in developing rats using electrophysiological recording techniques. In P5, P10, P15, P20 and adult rats, multiple recordings were made in the medial portion of S1 in each animal. Subsequently, these functional maps were related to anatomical parcellations of S1 based on a variety of histological stains. We found that at early postnatal ages (P5) medial S1 was composed almost exclusively of the representation of the vibrissae. At P10, other body part representations including the hindlimb and forelimb were present, although these were not topographically organized. By P15, a clear topographic organization began to emerge coincident with a reduction in receptive field size. By P20, body maps were adult-like. This study is the first to describe how topography of the body develops in S1 in any mammal. It indicates that anatomical parcellations and functional maps are initially incongruent but become tightly coupled by P15. Finally, because anatomical and functional specificity of developing barrel cortex appears much earlier in postnatal life than the rest of the body, the entire primary somatosensory cortex should be considered when studying general topographic map formation in development.

## Introduction

**Figure 1:**
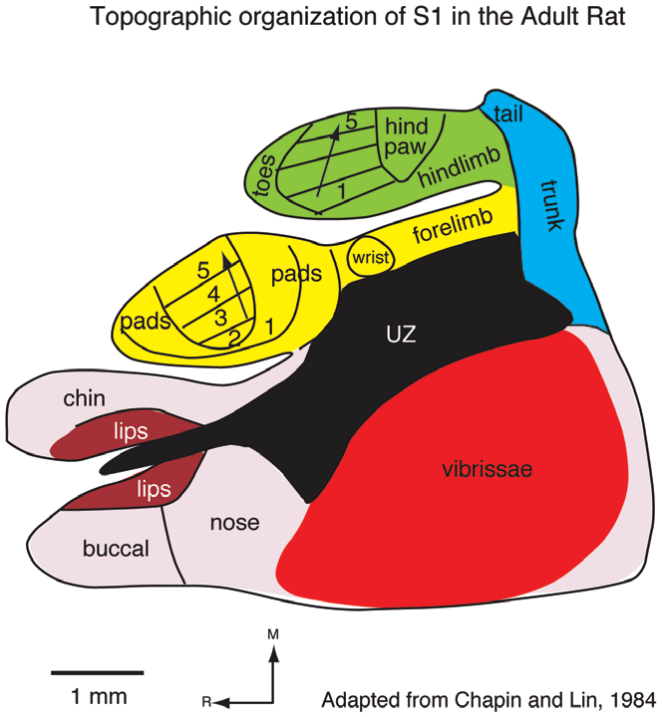
The topographic organization of the primary somatosensory area in adult rats. As in all other mammals examined, the contralateral body is represented from hindlimb to forelimb to face in a mediolateral progression. The individual toes of the hindpaw and digits of the forepaw are represented rostrally, the proximal limbs caudal to this and the trunk most caudally. In rats, there is a large magnification of the vibrissae of the face. In this and following figures, the head representation is shaded red, the forelimb representation is yellow, the hindlimb representation is green, and the trunk representation is blue. In this figure, unresponsive zones (UZ) are represented in black. Modified from Chapin and Lin, 1984.

Like all mammals, rats have a six-layered neocortex that can be divided into multiple functionally and anatomically defined areas, including three primary sensory areas: primary somatosensory cortex (S1), primary visual cortex (V1), and primary auditory cortex (A1; see Table 1 for abbreviations). In adult rats, S1 is the largest sensory area [1] and is somatotopically organized, with the tail representation located most medially and the nose and vibrissae representations located most laterally (Fig. 1) [2]. Although all portions of the contralateral body are represented within S1, different body part representations do not necessarily scale with the size of the body part itself, but rather with the use and innervation density of a body part [3]–[5]. For example, although the whiskers comprise a small portion of the body surface, they are highly innervated and, in rats, serve as the main effectors for sensorimotor exploration; consequently S1 is dominated by the representation of the vibrissae [6]–[8]. The topography of this whisker representation, or barrel field, is very precise in that there is a one-to-one correspondence between individual whiskers and architectonically defined barrels in the cortex, and this relationship persists even when differences in whisker pattern are generated in different strains [3].

**Table 1:** List of Abbreviations.

**Stains**	
5-HT	serotonin
AChE	acetylcholinesterase
CO	cytochrome oxidase
MBP	myelin basic protein
**Cortical areas**	
A1	primary auditory area
S1	primary auditory area
V1	primary visual area
V2	second visual area
DLCS	dorsolateral cortical sheet
VPm	ventral posteromedial nucleus
VPl	ventral posterolateral nucleus
LH	left cortical hemisphere
RH	right cortical hemisphere
SCtx	subcortical regions
**Ages**	
P5	postnatal day 5
P10	postnatal day 10
P15	postnatal day 15
P20	postnatal day 20
**Other Terms**	
ANOVA	Analysis of Variance
DAB	diaminobenzidine
DiI	1,1′-dioctadecyl-3,3,3′,3′-tetramethylindocarbocianine perchlorate
IM	intramuscular
IP	interaperitoneal
NGS	normal goat serum
PBS	phosphate buffered saline
rf	receptive field
NCR	no clear response
Res	responded to somatosensory stimulation

How the correspondence between peripheral patterns of receptors and cortical maps becomes established, and the role of intrinsic genetic mechanisms and extrinsic sensory driven activity in the linking of architectonic and functional maps, has been a long standing question in neuroscience. [9]–[13]. In rodents, the development and maturation of this close neuroanatomical and functional relationship has been almost exclusively studied for the whiskers of mice and rats, with little to no attention paid to how other portions of the body become functionally and topographically organized and related to architectonic distinctions. The two studies that do examine the development of topographic maps of the body either examine only a few developmental time points, or survey a very small portion of the entire S1 in any given animal [14], [15].

**Figure 2:**
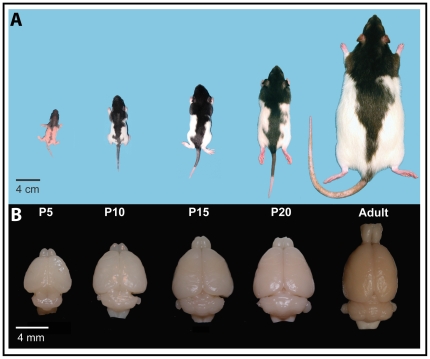
Changes in brain and body size across development. A) Scaled pictures of Long Evans rats at P5, P10, P15, P20, and during adulthood. B) The relative sizes of the rat brains at the same ages. While both the size and shape of the body change dramatically across development, the size and shape of the brain changes less significantly than the body.

The development of topographic maps in rats is especially interesting due to the tremendous changes that occur in both the brain and body during the early postnatal period. During the first three postnatal weeks, a rat's weight quadruples, and the size, shape, and orientation of its body changes dramatically (Fig. 2). Behaviorally, rats progress through several different stages of locomotion, beginning with punting at P4-5 (i.e., rotating their body by pushing with one forelimb) then quadrapedal crawling at P10-11, then walking at P12-13, and finally running by P15 [16], [17]. During this time, other motor skills begin to develop as well. Certain patterns of grooming can be identified as early as P2 [18]. Placing (i.e., lifting a paw and placing the sole flat against a test surface following stimulation of the dorsal paw) is observed in the forelimb by the end of the first postnatal week and in the hindlimb by the end of the second postnatal week [19], [20]. Other more complex skills, such as rearing, rope climbing, jumping, and object manipulation are observed by the end of the third postnatal week [17], [21]. As adults, rats are capable of executing highly dexterous paw and forelimb movements, especially during feeding and grooming [18], [22]–[24].

These physical and behavioral changes are accompanied by equally dramatic neural changes. While neurogenesis is largely complete by the day of birth, the process of neuronal migration proceeds well into the first postnatal week [25]–[27]. For example, neurons are still migrating into cortical layers 2 and 3 as late as P5-6 [28]. During this time, cortical neurons are experiencing a burst of exuberant synaptogenesis followed in the third postnatal week by synapse elimination and axonal pruning. During the first two postnatal weeks, layer specific thalamocortical connections are formed and thalamocortical synapses undergo a series of activity dependent changes, including long term potentiation and depression [29]–[34].

There are also significant neurochemical changes occurring during early postnatal development. The most dramatic and well known is the transition of gamma-amino butyric acid (GABA) from an excitatory neurotransmitter to its adult function as an inhibitory neurotransmitter [35]. This transition occurs during the second postnatal week [36] and is believed to be due to the down regulation of NKCC1 and the complementary up regulation of KCC2 [37], [38].

Thus, during the first three postnatal weeks, both the body and the brain undergo significant structural, biochemical and organizational changes, most of which have been documented almost exclusively within the whisker/cortical barrel system. Although the whisker/barrel field relationship has served as an important model for map formation and plasticity, it may not be the best model for all questions regarding map development. First, and perhaps most significantly, the barrel field is a highly derived feature of S1 that is only present in a subset of rodents, and is not representative of all rodents, and certainly not representative of mammals in general [7], [39]–[41]. In fact, it has been postulated that barrels may be an epiphenomenon associated more with the size of the brain than the presence of whisking [7]. Second, the anatomical pathways for the S1 representations of the head and body are different (trigeminal vs. dorsal column/cuneate, respectively) and developmental characteristics established for one may not generalize to the other.

In the present investigation, we examined the development of topographic maps of the body representation in rat S1 using electrophysiological recording techniques combined with histological analysis in P5, P10, P15, P20 and adult rats (see Table 2 for subject information). We had three goals. The first was to determine the types of histological markers that best reveal the cortical boundaries of S1 at different developmental stages. The second was to quantify the anatomical changes in the size of the overall brain and the proportion of the brain that S1 occupies throughout early to mid postnatal development. The final and most important goal was to examine the developmental time course of the emergence of topography of body maps and how strictly these maps correspond to the architectonic parcellations of S1, which are tightly coupled with maps of the body in adults.

**Table 2:** Subject Information.

Case #	Age	Sex	BodyWeight (g)	Experimental condition
08-143	P5	Male	13.8	Electrophysiol/Neuroanat
09-108	P5	Male	14.6	Neuroanatomy
09-109	P5	Female	12.4	Neuroanatomy
09-135	P5	Male	12.7	Neuroanatomy
10-026	P5	Male	12.1	Neuroanatomy
10-036	P5	Male	12.7	Electrophysiol/Neuroanat
10-125	P5	Female	15.5	Electrophysiol/Neuroanat
09-111	P10	Male	26.25	Neuroanatomy
09-112	P10	Female	26.4	Neuroanatomy
09-136	P10	Male	24.9	Electrophysiol/Neuroanat
10-030	P10	Female	24	Electrophysiol/Neuroanat
10-040	P10	Male	24.5	Electrophysiol/Neuroanat
10-164	P10	Female	28.5	Electrophysiol/Neuroanat
09-113	P15	Female	42.7	Neuroanatomy
09-114	P15	Male	43.9	Neuroanatomy
09-137	P15	Female	33.2	Electrophysiol/Neuroanat
09-145	P15	Female	33.5	Neuroanatomy
09-146	P15	Female	36.8	Electrophysiol/Neuroanat
10-035	P15	Female	33.9	Electrophysiol/Neuroanat
10-171	P15	Male	43.0	Electrophysiol/Neuroanat
09-029	P20	Female	66.0	Electrophysiol/Neuroanat
09-116	P20	Male	53.7	Neuroanatomy
09-117	P20	Female	55.5	Neuroanatomy
09-138	P20	Male	45.2	Electrophysiol/Neuroanat
09-148	P20	Male	49.1	Electrophysiol/Neuroanat
08-139	Adult	Male	275	Electrophysiol/Neuroanat
09-012	Adult	Female	275	Electrophysiol/Neuroanat
10-012	Adult	Male	360	Electrophysiol/Neuroanat
10-119	Adult	Female	325	Electrophysiol/Neuroanat

## Results

### Gross brain and body weight changes

Throughout development the rat body undergoes remarkable changes (Fig. 2A). Rats are altricial, and at birth weigh approximately 5 g. They are furless, their eyes and ears are sealed, they have limited thermoregulatory and locomotor capabilities and their skull and many other bones are uncalcified. Their head is disproportionately large and their limbs and tail are disproportionately short. Between postnatal days 10 and 15 they grow fur, their eyes and ears open, they begin to locomote independently, and they begin the process of weaning. By P20, rats are fully furred, capable of independent locomotion, are eating solid food, have well-developed visual and auditory systems, and have quadrupled in weight. However, as can be seen in Fig. 2A, they undergo a large amount of growth before their adult size is attained. While most studies have focused on the mystacial vibrissae, these structures undergo very little postnatal change compared to other body parts. For example, at birth the mystacial vibrissae in mice and other rodents exhibit the same grid-like arrangement that is seen in adult mystacial vibrissae [42], [43], and differentiation and maturation of sensory receptors is complete by the third postnatal week [44].

**Figure 3:**
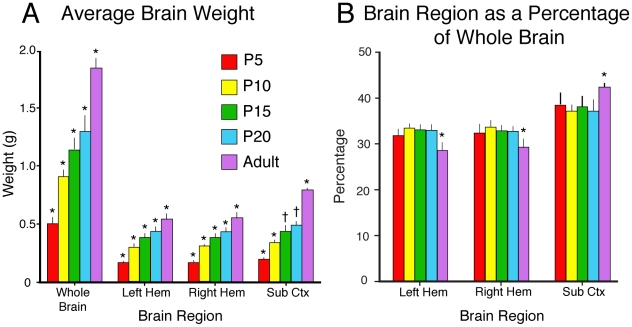
Changes in brain weight across development. A) Changes in the weight (in grams) of the whole brain, left hemisphere, right hemisphere, and subcortical regions across development. B) Changes in the weight of the left hemisphere, right hemisphere, and subcortical regions as a percentage of the weight of the whole brain across development. * - significantly different from all other ages. † - significantly different from P5, P10, and adult. Mean + s.d.

Like the body, the brain undergoes changes in size (Fig 2B; Table 3). The weight of the whole brain (including both cortical hemispheres, the thalamus, hypothalamus, cerebellum and brainstem) significantly increases across development (F_4,21_ = 130.44, *p*<.0001; Fig 3A). Likewise, the weight of each hemisphere (including the cortical sheet, olfactory bulb, pyriform cortex, hippocampus, and basal ganglia) significantly increases across development (F_4,21_ = 92.76, *p*<.0001; Table 3; Fig. 3A). The weight of the remaining brain sections (including thalamus, hypothalamus, brainstem, and cerebellum) significantly increases across development as well (F_4,21_ = 160.87, *p*<.0001; Table 3; Fig. 3A). Brain volume measurements (data not shown) follow a similar pattern.

**Table 3:** Brain Weights in grams.

Case #	Age	Whole brain	Left cortical hemisphere	Right cortical hemisphere	Subcortical structures	% LH	% RH	% SCtx
08-143	P5	-	-	-	-	-	-	-
09-108	P5	0.430	0.143	0.148	0.182	33.3	34.5	42.4
09-109	P5	0.542	0.162	0.162	0.189	29.9	29.9	34.9
09-135	P5	0.429	0.138	0.146	0.165	32.2	33.9	38.5
10-026	P5	0.502	0.159	0.167	0.195	31.7	33.2	38.9
10-036	P5	0.562	0.183	0.179	0.214	32.6	31.9	38.0
10-125	P5	0.542	0.179	0.180	0.213	33.0	33.2	39.3
**mean**		**0.501**	**0.161**	**0.164**	**0.193**	**32.1**	**32.8**	**38.7**
*s.d.*		*0.059*	*0.018*	*0.015*	*0.019*	*1.22*	*1.63*	*2.43*
09-111	P10	0.847	0.293	0.282	0.311	34.6	33.3	36.7
09-112	P10	0.838	0.279	0.283	0.321	33.3	33.8	38.3
09-136	P10	0.898	0.299	0.301	0.327	33.3	33.5	36.4
10-030	P10	0.914	0.299	0.299	0.333	32.7	32.7	36.4
10-040	P10	0.926	0.310	0.307	0.360	33.5	33.1	38.9
10-164	P10	1.027	0.354	0.373	0.397	34.4	36.3	38.6
**mean**		**0.908**	**0.306**	**0.307**	**0.341**	**33.6**	**33.8**	**37.5**
*s.d.*		*0.068*	*0.026*	*0.034*	*0.032*	*0.71*	*1.28*	*1.19*
09-113	P15	1.123	0.383	0.384	0.432	34.1	34.2	38.4
09-114	P15	1.107	0.379	0.382	0.455	34.3	34.5	41.1
09-137	P15	1.121	0.375	0.365	0.401	33.4	32.6	35.8
09-145	P15	1.059	0.359	0.345	0.382	33.9	32.6	36.1
09-146	P15	1.078	0.359	0.347	0.411	33.3	32.2	38.2
10-035	P15	1.135	0.359	0.379	0.472	31.6	33.4	41.5
10-171	P15	1.371	0.460	0.446	0.516	33.5	32.5	37.6
**mean**		**1.142**	**0.382**	**0.378**	**0.438**	**33.4**	**33.1**	**38.4**
*s.d.*		*0.105*	*0.036*	*0.034*	*0.046*	*0.89*	*0.91*	*2.23*
09-029	P20	-	-	-	-	-	-	-
09-116	P20	1.255	0.413	0.417	0.493	32.9	33.3	39.3
09-117	P20	1.275	0.421	0.416	0.503	33.0	32.6	39.4
09-138	P20	1.492	0.491	0.479	0.522	32.9	32.1	35.0
09-148	P20	1.181	0.410	0.403	0.427	34.7	34.1	36.1
**mean**		**1.301**	**0.434**	**0.429**	**0.486**	**33.4**	**33.0**	**37.5**
*s.d.*		*0.134*	*0.038*	*0.034*	*0.041*	*0.90*	*0.86*	*2.24*
08-139	Adult	1.820	0.522	0.565	0.776	28.7	31.0	42.6
09-012	Adult	-	-	-	-	-	-	-
10-012	Adult	1.936	0.591	0.583	0.818	30.5	30.1	42.3
10-119	Adult	1.819	0.505	0.507	0.791	27.7	27.9	43.5
**mean**		**1.858**	**0.539**	**0.552**	**0.795**	**29.0**	**29.7**	**42.8**
*s.d.*		*0.067*	*0.046*	*0.040*	*0.021*	*1.41*	*1.64*	*0.64*

The weights of the left hemisphere (LH), right hemisphere (RH), and subcortical structures (SCtx) were divided by the weight of the whole brain to determine the % LH, % RH, and % SCtx, and an ANOVA was performed to examine changes across development (Fig 3B; Table 3). The relative size of both the left and right hemispheres significantly decreased across development, but this effect was almost entirely driven by the adult values (% LH: F_4,21_ = 13.59, *p*<.05; % RH: F_4,21_ = 5.03, *p*<.05; Table 3). In contrast, the relative size of subcortical structures significantly increased across development (F_4,21_ = 4.14, *p*<.05), but as before the effect was almost entirely driven by the adult value. Thus, the relative size of cortical and subcortical structures remain constant from P5 through P20, but between P20 and adulthood the % LH and % RH significantly decrease while the % SCtx significantly increases.

### Cortical architecture and areal measurements of cortical areas

**Figure 4:**
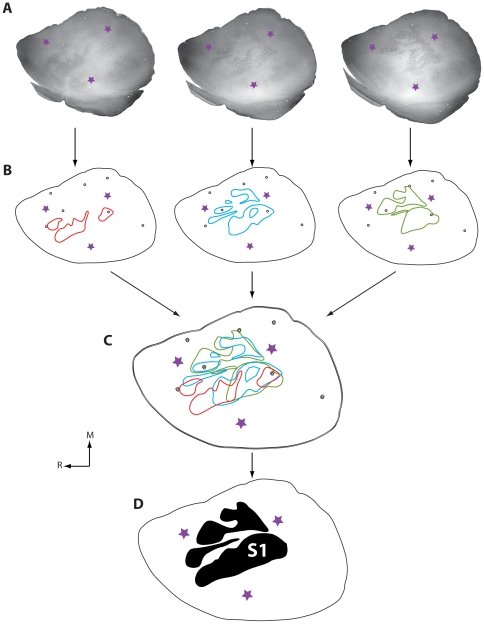
Reconstruction of architectonic borders. A) Photomicrographs are consecutive sections of CO-stained tissue each containing portions of the architectonic boundaries of the primary somatosensory area. Blood vessels appear as small white circles, and the primary sensory areas are visible as more darkly staining areas. The purple stars in all figures represent location of fluorescent probes placed in the brain during electrophysiological recording experiments and marked on the digital photograph of the brain, along with recording sites. By aligning these probes with digital images containing electrophysiological recordings, functional maps of S1 can be accurately matched to cortical architecture. B) The outer boundary of the cortical sheet and blood vessels are drawn along with portions of S1 (red, blue and green lines) that are visible in each section. C) By aligning blood vessels, data from all sections are superimposed onto a single section, and then a single comprehensive reconstruction of architectonic boundaries is drawn (D). In all sections, medial is to the top and rostral is to the left.

**Figure 5:**
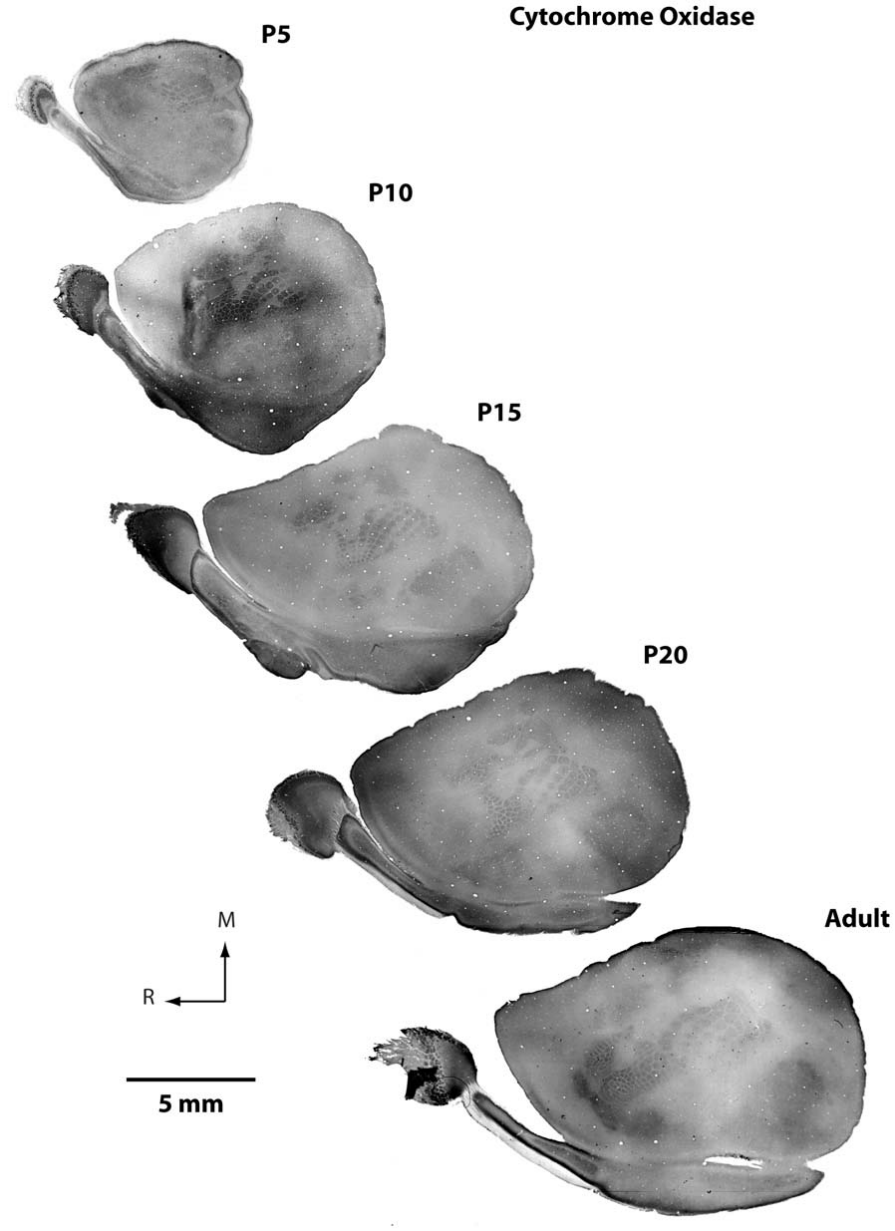
Appearance of cytochrome oxidase stained cortical tissue across development. Photomicrographs of cortex that has been flattened, sectioned tangentially and then stained for CO in P5 (top) through adult (bottom) subjects. Portions of the primary sensory areas, especially somatosensory cortex, are visible in all sections, but individual sections do not show all of the boundaries of a field. Images were imported into Adobe Photoshop and levels were adjusted to increase contrast. In all photomicrographs, medial is to the top and rostral is to the left.

**Figure 6:**
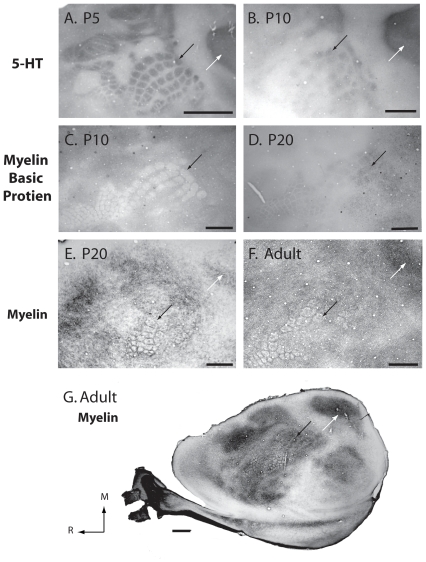
Differential effectiveness of cortical staining techniques. Photomicrographs of primary somatosensory cortex in 5-HT- (A and B), MBP- (C and D), and myelin-stained sections (E, F, and G) in P5 (A), P10 (B and C), P20 (D and E), and adult (F and G) subjects. Visible barrels are indicated with black arrows, and V1 is indicated with white arrows. Different stains work best at different ages. Images were imported into Adobe Photoshop and levels were adjusted to increase contrast. In all photomicrographs, medial is to the top and rostral is to the left. See Table 1 for abbreviations. Scale bar = 1 mm.

We used several different histological techniques to identify the boundaries of cortical fields at different ages (see Fig. 4 for an explanation of cortical field identification). This was necessary because, with the exception of cytochrome oxidase (CO; Fig. 5), different stains worked optimally at different ages (Fig. 6). Because cortex was cut tangential to the cortical surface to aid in accurately relating different histological stains to electrophysiological recordings, we were unable to precisely localize the laminar differences in staining. However, we have made distinctions such as superficial, middle and deep. At all ages examined, S1 was consistently identified as darkly staining for CO, especially in middle layers. The pattern of CO staining in S1 was heterogeneous with islands of lightly stained tissue surrounding the dark regions, clearly delineating what would be different body part representations in the adult (see Figs. 1 and 4). Such a pattern has been previously described for P5, P10, P15, P21 and adult rats [45], [46]. The large, lateral darkly staining region contained the posteriormedial barrel subfield representing the vibrissae in adults. Additional dark islands interspersed by small non-staining bands separated the chin representation from the forelimb, hindlimb and trunk representations as previously described in the adult. While CO exhibited a consistent staining pattern across all ages, our electrophysiological recording results demonstrate for the first time that the electrophysiologically-determined somatotopic organization that is co-extensive with this pattern in the adult is not consistently present until P20 in developing rats (see below).

We also used serotonin (5-HT), myelin basic protein (MBP) and myelin stains and related these patterns to CO staining and electrophysiological recordings at different postnatal ages (Fig. 6). As with CO staining, at P5, 5-HT clearly marked a number of cortical areas including S1, A1, and V1 and was darkest in the middle cortical layers. The pattern of staining was almost identical to cytochrome oxidase in that S1 stained heterogeneously and large non-staining zones demarcated separate divisions, while smaller non-staining bands marked small islands within these zones (Fig. 6A). V1 and A1 were darkly and homogeneously staining as described previously in early postnatal rats and mice [47]–[49]. This pattern persisted at P10, but was somewhat less distinct, especially within S1 (Fig. 6B). By P15 we were no longer able to identify any boundaries using 5-HT.

While 5-HT failed to demarcate boundaries past P10, we had moderate success defining cortical field boundaries using MBP staining, which, at certain developmental stages, resulted in differential patterns of staining in middle cortical layers (Fig. 6C and D). At P10 only the barrel fields of S1 could be demarcated (Fig. 6C). Unlike CO and 5-HT staining, the barrels were unstained while the peri-barrel region was darkly stained, thus appearing as a negative of the CO and 5-HT patterns. At P15 a similar pattern of MBP was produced for the barrels of S1. However, at P20, the pattern was altered in that the barrels stained darkly and the peri-barrel region lightly, much like the pattern of staining produced by CO (Fig 6D). As with early aged animals, staining was only apparent within the posteromedial barrel subfield of S1. We were unable to successfully stain brains younger than P10 or older than P20 with MBP

In late postnatal animals and adults, myelin stains clearly delineated cortical fields boundaries. Although these stains were not successful in delineating cortical fields of animals younger than P20, by this postnatal age a clear pattern of myelin staining emerged that looked much like that described in this and previous studies in adult rats (Fig. 6E–G) [1], [50], [51]. As in adults, the pattern of staining was most distinct in middle cortical layers. S1 stained darkly but heterogeneously for myelin, with the barrel cortex forming a negative of the CO pattern. Thus, peri-barrel regions were stained, similar to the pattern observed for MBP at P10 and P15. Other portions of S1 that in adults represent the body and limbs stained darkly and homogenously for myelin (Fig. 6G). When the entire series of sections was examined, it could be seen that these major body parts were separated by lightly myelinated zones.

**Figure 7:**
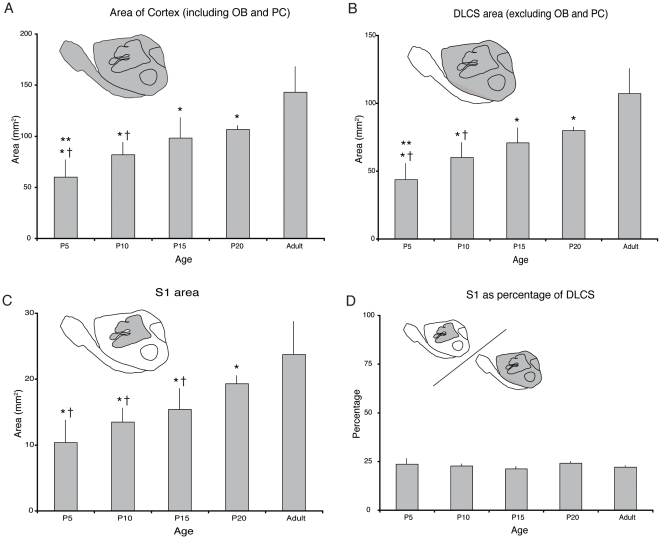
Measurements of different cortical areas. The shaded portion of the brain schematic represents the area being measured. A) The mean area (in mm^2^) of the cortex (including the dorsolateral cortical sheet, pyriform cortex, and olfactory bulb), B) dorsolateral cortical sheet (DLCS), (C) S1, and (D) the percentage of cortex occupied by S1. Note that the size of the complete cortical sheet, DLCS and S1 increases with age. However, the relative size of S1 as measured using architectonic boundaries is unchanged throughout development and adulthood. * - significantly different from adult. † - significantly different from P20. ** - significantly different from P15. Mean + s.d.

Using these various patterns of staining we measured the area of a number of compartments within the developing and adult brain and found a consistent relationship across regions (Fig. 7; Table 4). Measurements of the dorsolateral cortex (including the olfactory bulb and pyriform cortex) across age groups indicate that its area significantly increases across development (F_4,13_ = 12.94, *p*<.0005; Fig. 7A and Table 4). A similar pattern of change is observed when only the dorsolateral cortical sheet is measured across age groups (F_4,13_ = 15.92, *p*<.0001; Fig. 7B and Table 4) suggesting that the gross morphology of the cortex changes size at a similar rate. When the absolute size of S1 is measured across age groups we observed that it, too, significantly increases in size across development (F_4,13_ = 11.17, *p*<.0005; Fig. 7C and Table 4). Finally, when the size of S1 is calculated as a percentage of the DLCS we find that there is no change in the relative size of this field across different ages (F_4,13_ = 1.88, *NS*; Fig. 7D and Table 4).

**Table 4:** Brain Areas in mm^2^.

Case #	Age	Ctx	DLCS	S1	% S1
08-143	P5	52.547	37.174	9.686	26.1
10-036	P5	79.311	57.507	14.063	24.5
10-125	P5	48.223	36.554	7.457	20.4
**mean**		**60.027**	**43.745**	**10.402**	**23.6**
*s.d.*		*16.840*	*11.922*	*3.361*	*2.9*
09-136	P10	96.115	71.976	15.769	21.9
10-030	P10	69.196	46.072	10.670	23.2
10-040	P10	87.126	64.431	13.878	21.5
10-164	P10	75.221	58.250	13.616	23.4
**mean**		**81.915**	**60.182**	**13.483**	**22.5**
*s.d.*		*12.047*	*10.954*	*2.107*	*0.9*
09-137	P15	104.850	73.992	16.783	22.7
09-146	P15	81.634	60.373	12.589	20.9
10-035	P15	82.484	64.273	13.029	20.3
10-171	P15	123.593	84.956	19.226	22.6
**mean**		**98.140**	**70.899**	**15.407**	**21.6**
*s.d.*		*20.087*	*10.983*	*3.166*	*1.2*
09-029	P20	108.970	82.282	20.173	24.5
09-138	P20	102.241	77.165	17.912	23.2
09-148	P20	108.654	80.265	19.834	24.7
**mean**		**106.622**	**79.904**	**19.306**	**24.1**
*s.d.*		*3.797*	*2.578*	*1.219*	*0.8*
08-139	Adult	169.409	121.582	26.792	22.0
09-012	Adult	168.647	127.881	29.448	23.0
10-012	Adult	141.865	101.132	21.799	21.6
10-119	Adult	118.350	92.618	20.054	21.7
**mean**		**149.568**	**110.803**	**24.523**	**22.1**
*s.d.*		*24.438*	*16.654*	*4.351*	*0.7*

Taken together, our histological analysis and cortical measurements revealed several important findings. The first is that CO staining can be used throughout development to identify what will become the functionally defined S1 in adults. Second, this CO pattern is consistent throughout development and its size relative to the dorsolateral cortical sheet (DLCS) remains constant throughout the lifespan. Third, other stains work well for identifying S1, but only in animals of a particular age. 5-HT works well in the cortex of early postnatal rats (P5-P10), MBP works well during middle stages of development (P10–P20) and myelin stains work well at later stages of development and in adults. Fourth, all of these stains, especially in different combinations at different ages, can be used to accurately mark cortical field boundaries, but the match between functional organization and cortical architecture is imprecise. This relationship is described in the following section. Fifth, aspects of brain organization such as overall area of the cortex, area of DLCS and size of S1, change in a consistent manner across age groups, while some aspects of organization, specifically the percentage of cortex occupied by S1, remain consistent. Finally, as illustrated in Figure 2, the brain and body grow at very different rates. Although the brain gradually increases in size from birth through adulthood (from 0.50±0.05 g at P5 to 1.86±0.07 g in adults), it does so in a relatively uniform manner, with the cortical hemispheres increasing in weight at approximately the same rate as the subcortical structures. In contrast, the body undergoes a much more dramatic increase in size across the same time period (from 13.40±1.27 g at P5 to 308.75±41.51 g in adults), and different parts grow at different rates. For example, at P5, the head comprises about one third of the length of the body, while in adults the head comprises less than half that amount.

### The development of gross topographic organization within S1

**Figure 8:**
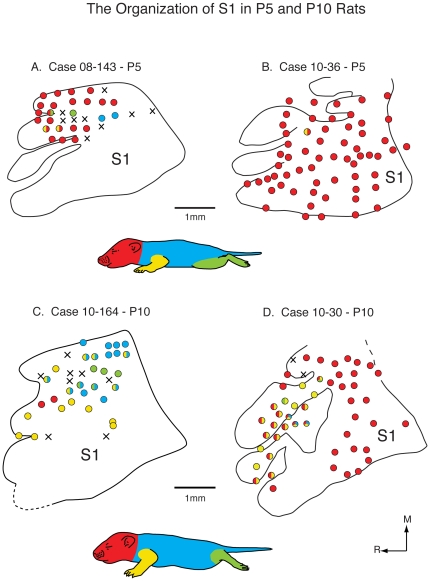
Functional S1 maps in P5 and P10 rats. Reconstructed functional maps of primary somatosensory cortex in P5 (A and B) and P10 (C and D) rats. A schematic of a rat body is divided into different major sections: the head, portions of the face and the vibrissae are red, the trunk and tail are blue, the forelimbs are yellow, and the hindlimbs are green. Recording sites that contained neurons that had receptive fields on these major parts are shown as circles filled with that color (i.e., yellow). Recording sites that contained neurons that responded to multiple body parts are labeled with the colors of all of the corresponding body parts. Recording sites that did not respond to any somatosensory stimulation are marked with an X. At P5, medial portions of S1 that normally represent portions of the limbs and trunk contain representations of the face/vibrissae with only a few sites containing neurons responsive to stimulation of the contralateral body. By P10, representations of body parts are beginning to emerge. Conventions as in previous figures.

**Figure 9:**
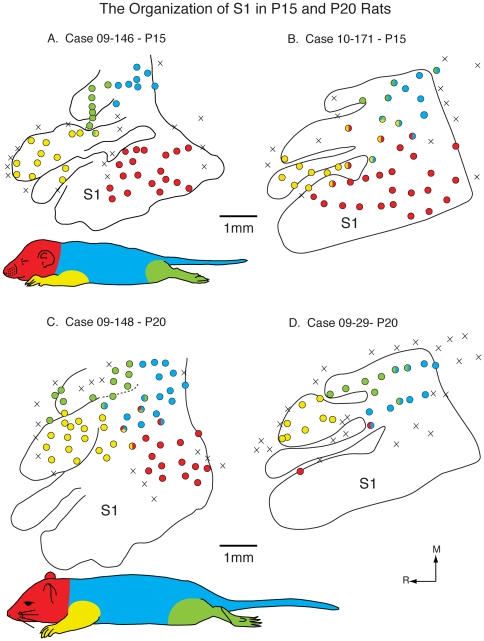
Functional S1 maps in P15 and P20 rats. Reconstructed functional maps of primary somatosensory cortex in P15 (A and B) and P20 (C and D) rats. At P15, normal somatotopy is beginning to emerge, but there is still considerable variability in map organization between individuals (compare A and B). By P20, the functional maps have an adult-like organization. Conventions as in previous figures.

**Figure 10:**
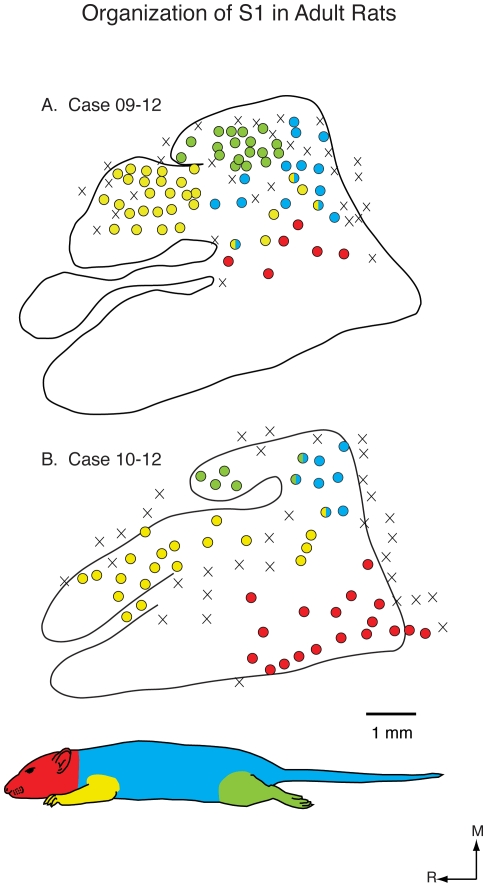
Functional S1 maps in adult rats. Reconstructed functional maps of primary somatosensory cortex in adult rats (A and B). In adult rats the topographic organization is precise and consistent across animals and similar to that previously described (Chapin and Lin, 1984). Conventions as in previous figures.

Multiple recording sites (Table 5) in the medial portion of what would become the body representation of S1 in adults were made in P5, P10, P15, P20 and adult animals (Figs. 8, 9, 10). As the organization of the posteromedial barrel subfield is well documented throughout development, we did not endeavor to obtain a complete map of that region. Our goal in these experiments was to examine how the entire body representation within S1 changes across development, and how precisely it corresponds to architectonic distinctions. In the first section of these results we describe the overall topographic organization of cortex that is coextensive with architectonically defined S1. We then show receptive field size and progression across the architectonically defined body representation within S1. Finally we show neural activity at selected sites and the receptive fields of neurons at these sites. In order to get a general appreciation of the topography of S1, we subdivided the developing body into 4 major segments which include: 1) The entire face, head, neck, pinna, and vibrissae, 2) the forelimb up to the shoulder, 3) the entire trunk and tail, and 4) the hindlimb up to the hip (see animal bodies in Figs. 8, 9, 10). We did this to more easily and clearly visualize the changes in gross somatotopic organization across development.

**Table 5:** Recording Sites.

Case No.	Age	Head	Forelimb	Trunk	Hindlimb	Mixed	NCR	Res	TOTAL
08-143	P5	18	0	2	2	2	9	24	**33**
10-036	P5	59	0	0	0	1	0	60	**60**
10-125	P5	24	4	1	0	3	13	31	**44**
09-136	P10	9	1	4	0	2	13	16	**29**
10-030	P10	28	4	0	0	17	2	49	**51**
10-040	P10	6	12	1	4	3	27	26	**53**
10-164	P10	2	13	14	4	0	12	33	**45**
09-137	P15	0	3	8	6	4	7	21	**28**
09-146	P15	19	12	7	9	0	18	47	**65**
10-035	P15	17	0	0	0	3	10	20	**30**
10-171	P15	23	10	7	4	1	13	45	**58**
09-029	P20	0	5	11	6	2	26	24	**50**
09-138	P20	15	3	13	4	1	14	36	**50**
09-148	P20	13	17	15	13	0	18	58	**76**
08-139	Adult	1	11	2	5	1	10	20	**30**
09-012	Adult	6	29	14	18	0	31	68	**99**
10-012	Adult	18	19	8	4	0	33	49	**82**
10-119	Adult	19	10	2	9	2	33	42	**75**
**TOTAL**		**277**	**153**	**109**	**88**	**42**	**289**	**669**	**958**

In P5 animals, we were able to obtain electrophysiological recordings predominantly in the medial portion of S1 in one animal (8A) and in both the medial and lateral portion of S1 in two animals (Fig. 8B and case 10–125, not shown). In these animals, most neurons had contralateral receptive fields, although in some cases, we observed bilateral receptive fields (Table 6). The most important observation was the preponderance of receptive fields on the face, predominantly the vibrissae in medial portions of S1 that normally represents portions of the body and limbs in adults. In one animal (Fig. 8B), we found only one receptive field on a body part other than the face and vibrissae. In the two other cases, there were a few recording sites with receptive fields exclusively on the hindlimb, trunk and/or forelimb (Fig. 8A) and a few recording sites with receptive fields on multiple major body parts, which we term “mixed” in Table 5.

**Table 6:** Laterality of Receptive Fields.

Case No.	Age	Contralateral	Ipsilateral	Bilateral	total
08-143	P5	4	10	10	**24**
10-036	P5	0	0	60	**60**
10-125	P5	28	1	2	**32**
09-136	P10	3	4	9	**16**
10-030	P10	25	0	24	**50**
10-040	P10	23	0	3	**26**
10-164	P10	24	2	7	**33**
09-137	P15	15	0	6	**21**
09-146	P15	47	0	0	**47**
10-035	P15	4	0	16	**19**
10-171	P15	41	0	4	**45**
09-029	P20	20	1	3	**24**
09-138	P20	29	0	7	**36**
09-148	P20	58	0	0	**58**
08-139	Adult	20	0	0	**20**
09-012	Adult	68	0	0	**68**
10-012	Adult	49	0	0	**49**
10-119	Adult	42	0	0	**42**
**TOTAL**		**500**	**18**	**151**	**670**

At P10, we recorded from neurons in S1 in four animals (Table 5; Fig. 8C and D), and while there was some variability in map organization, there was a clear difference when compared to maps generated at P5. The most notable difference was an increase in the number of recording sites in which neurons had receptive fields exclusively on portions of the hand, foot or trunk, or mixed receptive fields that incorporated multiple body parts other than the face or vibrissae. In some cases, receptive fields on the face/head were present in locations normally occupied by other body parts (e.g. Fig. 8C and D). Despite the emergence of neurons with receptive fields on the limbs and trunk, the topography was imprecise, and did not resemble that of an adult (see below). In two cases, at some recording sites there was correct localization of functionally defined forelimb representation within the architectonic zone that is co-extensive with the forelimb representation in adults (e.g. Fig. 10C). However, there were neurons with receptive fields on the forelimb that were well outside of the architectonically defined forelimb compartment.

At P15, electrophysiological recordings revealed that a topographic order had begun to emerge, and that functional maps of the body were better aligned with the architectonic compartments in S1 (Fig. 9A and B). Except for one case (10–35, not shown) there were few recording sites in medial portions of S1 that had neurons with receptive fields on the face/vibrissae. The number of exclusively localized receptive fields on the limbs or trunk increased and the number of mixed sites decreased. There was a clear topography with the forelimb representation located lateral to the hindlimb representation, the trunk representation located caudal to the limb representation, and the vibrissae/face representation located laterally within the architectonically defined S1. The few mixed receptive fields that were observed were associated with neurons at the interface between major body parts such as the limb/trunk representations. Further, the functional representations of different body parts, such as the forelimb and trunk, in most cases fit within the architectonically defined compartments associated with these body parts in the adult (e.g. Fig. 9A).

Maps generated in three P20 rats had a clear topography (Fig. 9C and D), and most recording sites contained neurons with receptive fields localized exclusively to a portion of a specific body part such as the forepaw, forelimb or trunk. The mediolateral and rostrocaudal topography was like that described in previous studies in adult rats (Fig. 10). Finally, the functionally defined maps of the limbs, trunk and face were coextensive with architectonically defined zones associated with these body parts in adults.

The maps of S1 in adults have been well described in previous studies [2], [8], [52]. Our maps in adults (Fig. 10A and B) are similar to those generated previously and serve as a comparison with the maps generated in developing animals using identical anesthetic, recording and histological methods, as well as techniques for data analysis. The functional maps in adults generated in this study show a clear topographic organization with the foot represented medial to the forepaw and the proximal limb and trunk represented caudal to the paws. The face was represented lateral to the representations of the limbs and body. Receptive fields for neurons were localized exclusively to specific portions of the body, with only a few mixed receptive fields occurring within portions of S1 that were transitions between major body part representations. As previously reported, the maps of portions of the body were co-extensive with architectonically defined zones within S1.

Taken together, our results on the development of topographic maps of the body in rats demonstrate that early in development topography is absent and most of S1, including those portions normally allocated to representing limbs and the trunk, is occupied by the representation of the vibrissae/face. Thus there is little or no correspondence between functional representation of the body and architectonic zones that is so remarkable in the adult. By P10, representations of other body parts begin to emerge, but their topography is imprecise, there is still an over-representation of the vibrissae, and there is little to no correspondence between architectonic and functionally defined zones within S1. By P15 a clear topography is present, and there is an apparent correspondence between architectonically defined zones within S1 and topographic maps. By P20, topographic organization is distinct and functional maps correspond well with architectonic maps and look similar, but not identical, to maps generated in the adult.

### Receptive field progressions and the development of receptive field configuration

**Figure 11:**
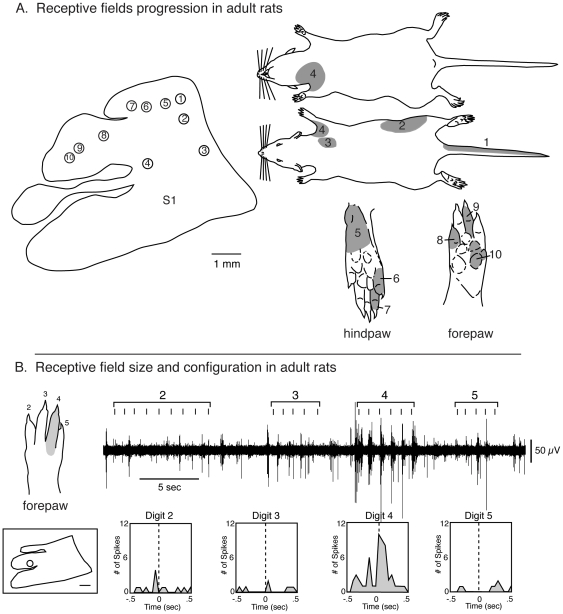
Receptive field progressions in an adult rat. Progressions of recording sites in S1 in an adult rat (left in A) and corresponding receptive fields for neurons at those sites (right in A). Numbered sites in the cortex correspond to numbered receptive fields on the body. Receptive fields are shaded grey. In adults, the topographic organization is precise and consistent across animals. As recording sites progress from medial to lateral in the caudal portion of S1 (sites 1–4) corresponding receptive fields move from the tail, lower trunk to upper trunk. The hindpaw (5–7) and forepaw (8–10) have corresponding progression from toes 5–1 and digits 5–1, respectively. Compare this figure with the full map of the body illustrated in Figure 1. B) Representative trace of cortical activity in response to stimulation of digits of the contralateral digit 4 (top left) in an adult rat. In the top left drawing of the forepaw, each digit is labeled with its corresponding number, and the receptive field on dorsal digit 4 is shaded in grey. In the bottom left is a schematic of S1 with the recording site marked with an open circle (scale = 1 mm). A trace of multi-unit activity is located to the right of the forepaw schematic. Tic marks represent the temporal pattern of stimulation. Peri-event histograms, labeled with their corresponding digit, show the increase in the amount of cortical activity in the 1 second surrounding digit stimulation.

In this portion of the results we first describe the receptive field progression and configuration of receptive fields observed in adults, and then describe similar progressions in animals of descending age. We describe this section in descending age to better illustrate the lack of topography in progressively younger animals. Finally we describe the receptive fields for neurons in similar anatomical parcellations of S1 from P5 through adulthood. The topography of S1 in adults has been well described in previous studies (Fig. 1) [2], [8], [52], and in the current investigation, receptive field progressions in different portions of S1 reveal the precise topography that has been previously reported. With recording site progressions from medial to lateral in the caudal portion of S1, where the trunk is represented, receptive fields at these sites progress from the dorsal tail, to the lower and then upper dorsal trunk (Fig. 11A rfs 1–3). The ventral trunk is represented rostrally (Fig. 11A, rf 4). As in previous studies, progressions from caudomedial to rostrolateral within the hindpaw representation produce receptive fields on the foot that move from T5 – T1 (Fig. 11A rf 5–7). A similar topography is observed for the forepaw with recording site progression from caudomedial to rostrolateral having corresponding representations on digits 5–1 (and pads) respectively (Fig. 11A rf 8–10). When single or multiple units are examined and quantified, and receptive field size strictly localized, as in previous studies, we find that receptive fields are localized to very small portions of the paws or body. For example, in the adult it is common for receptive fields on the forepaw to be limited to a small portion of a distal digit or a dorsal digit (Fig. 11B).

**Figure 12:**
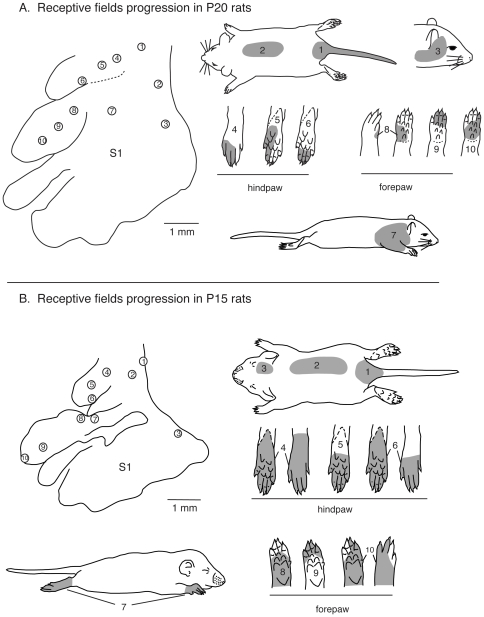
Receptive field progressions in P20 and P15 rats. A) Progressions of recording sites in S1 in a P20 rat (left) and corresponding receptive fields for neurons at those sites (right). In P20 rats, the topographic organization is similar to that seen in adults. As recording sites progress from medial to lateral in the caudal portion of S1 (sites 1–3) corresponding receptive fields move from the tail, hindlimb and lower trunk to upper trunk and face. Compared to adults, receptive fields on the hindpaw (4–6) and forepaw (8–10) are larger and can encompass multiple digits, toes, or pads. B) Progressions of recording sites in S1 in a P15 rat (left) and corresponding receptive fields for neurons at those sites (right). In P15 rats the topographic representation is less well-organized and there is greater variability between animals. Receptive fields are larger and can encompass more than one body part (i.e., site 7). As recording sites progress from medial to lateral in the caudal portion of S1 (1–3) corresponding receptive fields move from the tail and lower trunk, to the middle trunk and head. Most often receptive fields are on the entire foot (4–5) or large portions of the forepaw (7–10). Compare this figure with the full map of the body illustrated in Figure 1 [102]. Conventions as in previous figures.

In P20 rats, body part representations in S1 had a somatotopic organization that was similar to adult rats, with the hindlimb representation located most medially and the head and vibrissae representations located most laterally. To illustrate this, we examined recording sites in a similar progression as that in adults (Fig. 11). As in adults, in the caudal portion of S1 as recording sites move from medial to lateral, corresponding receptive fields for neurons at those sites progress from the tail and lower trunk to the upper dorsal trunk and then face (Fig. 12A rfs 1–3). Within the hindpaw representation, as recording sites progress from caudomedial to rostrolateral corresponding receptive fields generally progress from the lateral to medial toes and foot, however the receptive fields encompass multiple toes (Fig. 12A, rfs 4–6). Similarly, within the forepaw representation recording site progression from caudomedial to rostrolateral yields receptive fields that move from proximal forelimb (Fig. 12A, rf 7) to the forepaw, and on the forepaw progress from the lateral to medial portion of the hand and digits (Fig. 12A, rfs 8–10). However, the size of receptive fields was typically larger than in adults, often encompassing parts of multiple digits and toes.

In P15 rats, the organization of body part representations in S1 varied between cases. In some animals, S1 organization was relatively somatotopic, while in others the functional organization did not conform to the architectonic parcellations of S1 in adults (see Figs. 9A–B). In the case shown in Fig. 12B, as recording sites progressed from medial to lateral in the caudal portion of S1, corresponding receptive fields for neurons at those sites progressed from dorsal lower trunk and tail to upper trunk, to head (Fig. 12B rf 1–3). While the rostromedial portion of S1 contained neurons with receptive fields on the hindpaw, they were large, and there was no apparent topography (Fig. 12B rf 4–6). This was also true for the forepaw (Fig. 12B, rf 7–10, also see Fig. 14). The size of individual receptive fields was notably larger than in older rats, often encompassing large portions of the fore- and hindpaws (Fig. 12B, rf 5 and 8) or both dorsal and ventral surfaces (rfs 4, 6 and 10).

**Figure 13:**
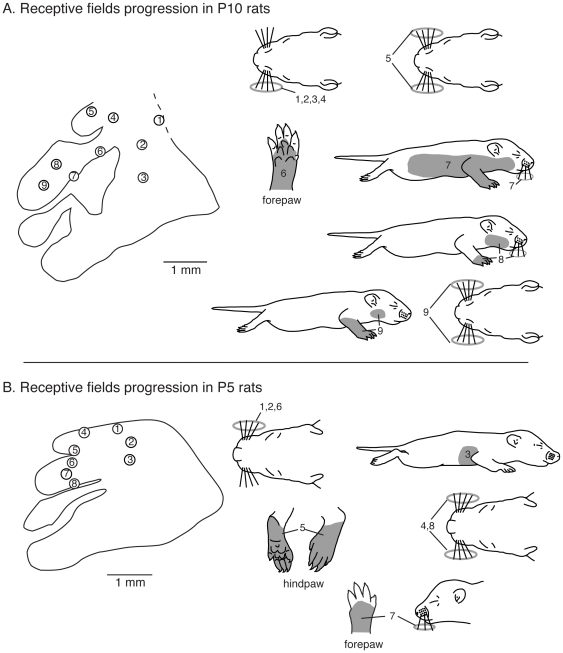
Receptive field progressions in P10 and P5 rats. A) Progressions of recording sites in S1 in a P10 rat (left) and corresponding receptive fields for neurons at those sites (right). In P10 rats, the topographic organization is imprecise. The receptive fields are very large and many receptive fields cover multiple body parts (i.e., sites 7–9). Vibrissae representations are found throughout S1 in inappropriate locations (i.e., sites 1, 2, 4 and 5). As recording sites progress from medial to lateral in the caudal portion of S1 (1–3) corresponding receptive fields were all on the ipsilateral vibrissae. Recording sites in the far medial location (4, 5), in what would be the hindpaw representation in the adult, had receptive fields on the ipsilateral or vibrissae. Recording sites in medial portions of S1 in what would normally be the forepaw representation (6–9) had receptive fields on the forepaw, split receptive fields on the upper body and vibrissae, bilateral vibrissae and face and vibrissae. B) Progressions of recording sites in S1 in a P5 rat (left) and corresponding receptive fields for neurons at those sites (right). In P5 rats there is no apparent topography. Receptive fields are large, and, when present on the limbs, encompass both hairy and glabrous portions of the paws. Receptive fields are also observed on both the contralateral and ipsilateral body parts. Vibrissae representations are prevalent and found throughout S1. As recording sites progress from medial to lateral in the caudal portion of S1 (1–3) corresponding receptive fields move from the contralateral vibrissae to the lateral trunk. Far medial recording sites (4–5) in what would normally be the hindpaw representation had receptive field on the vibrissae, and in one instance the dorsal and ventral hindpaw. More medial recording sites (6–8), in what would normally be the forepaw representation had receptive fields on the contralateral or bilateral vibrissae, and wrist and vibrissae. Compare this figure with the full map of the body illustrated in Figure 1. Conventions as in previous figures.

In P10 rats, there was considerable variability across cases. However, in general, topography was imprecise or absent, and receptive fields for neurons did not uniformly conform to the architectonic borders of S1 (see Figs. 6C–D). For example, mediolateral progressions of recording sites in the caudal portion of S1 all contained neurons with receptive fields on the vibrissae (rather than on the trunk, e.g. Fig. 13A rfs 1–3). The medial portion of S1 that in adults contains neurons with receptive fields on the hindpaw had receptive fields on the vibrissae in this P10 animal (Fig. 13A rf 4–5). In what would normally be the forepaw representation just lateral to the hindpaw representation, there was no progressive topography, as is typically seen in adults. Only a few recording sites contained neurons with receptive fields on the forepaw (e.g. Fig. 13A, rf 6) and some of these (e.g. rfs 8 and 9) also had receptive fields on other body parts such as the vibrissae or face.

In P5 rats, all architectonic segments of S1 are dominated by the vibrissae, and when other body part representations were present, they showed no topography. The small islands of neurons with receptive fields on other body parts may or may not be in the appropriate somatotopic locations as described in adults. The receptive fields for neurons on non-vibrissae body parts were generally large (e.g. Fig. 13B rf 5), and while predominantly contralateral, we observed ipsilateral and bilateral receptive fields as well (e.g. Fig. 13B rfs 4, 7 and 8). Thus, although the anatomical parcellations of S1 in P5 rats appear nearly identical to those in adults, these parcellations do not correspond to a topographically organized map of the contralateral body, as they do in adults.

**Figure 14:**
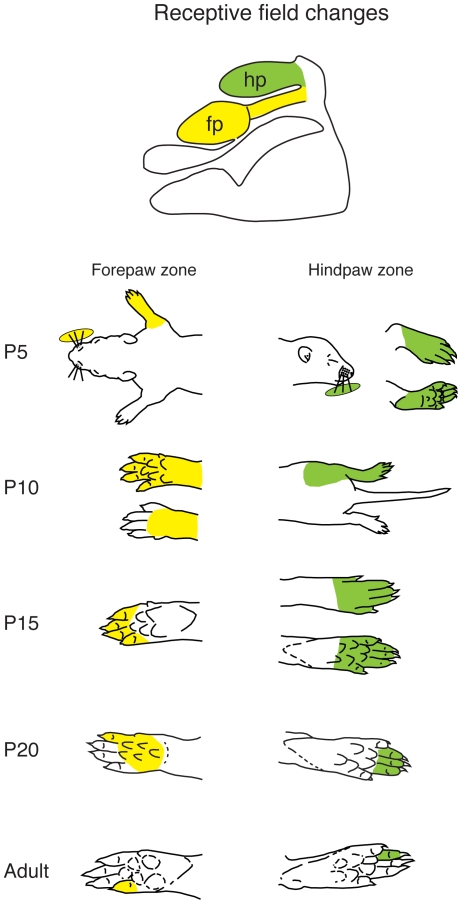
Changes in receptive field size and configuration at different developmental ages. The top illustration is a schematic of the body map in adults with the hindpaw zone marked in green and the forepaw zone marked in yellow. The receptive fields below for the different postnatal ages are for neurons in recording sites in each of these zones. At P5 there were very few recording sites with neurons that had receptive fields on either the forelimb or hindlimb. For those that did, receptive fields were large, and encompassed the vibrissae as well. With progressively older postnatal ages, the size of receptive fields for neurons in these zones decreased, and in adults, were small and often encompassed only a single digit or toe. Conventions as in previous figures.

**Figure 15:**
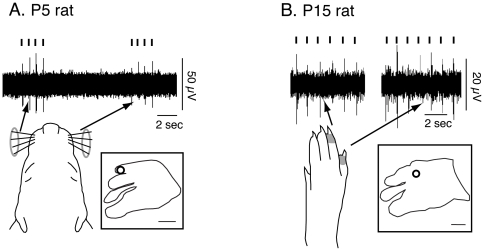
Representative traces of multi-unit activity in a P5 (A) and P15 (B) rat. A) Multi-unit activity in response to stimulation of both ipsilateral (left) and contralateral (right) vibrissae. Tic marks indicate the temporal pattern of stimulation. The inset box includes a depiction of S1 with the recording site indicated by an open circle (scale = 1 mm). B) Multi-unit activity in response to stimulation of toe 4 (left) and toe 5 (right) of the contralateral hindpaw. The receptive field for the neurons is indicated in gray on the schematic of the contralateral hindpaw. The inset box includes an illustration of S1 with the recording site marked by an open circle (scale = 1 mm).

To directly compare changes in receptive fields' size and configuration, we examined the receptive fields for neurons at recording sites in the same architectonic zones (anatomical parcellations) that in adults are coextensive with the hindpaw and the forepaw representations (Fig. 14). At P5, receptive fields for neurons in the far medial zone were predominantly on the contralateral vibrissae, with some on the ipsilateral or bilateral vibrissae (Fig. 15). For two sites in one animal we did find receptive fields on the entire foot, but the vibrissae were also included in this receptive field. At P5, for the anatomical parcellation that in the adult contains a representation of the forepaw, most of the recording sites in most of the animals had neurons with receptive fields on the vibrissae. At two sites in one case and seven sites in another case, we did observe receptive fields on the forelimb, but these were very large and included the entire forelimb and also encompassed the vibrissae (Fig. 14). At P10, neurons in the hindpaw and forepaw zones had receptive fields that were mostly restricted to the limb, but these were very large. There was variable representation of the vibrissae within these zones in different cases. With an increase in age, receptive fields for neurons in the hindpaw and forepaw zones became progressively smaller, were localized to a single body part and did not include the vibrissae (Fig. 14).

Although the receptive fields and body part representations within S1 change dramatically across development, the neuronal responses to somatosensory stimulation can be quantified at all ages (Fig. 15). Figure 15A shows the neural response in a P5 rat to bilateral stimulation of the vibrissae, while 15B shows the neural response in a P15 rat to stimulation of the contralateral T4-5. Although the amount of cortical activity [53], [54] and latency of response [55] changes across development, in all cases driven neuronal responses to somatosensory stimulation was clear, discrete, and easily distinguishable from background activity.

## Discussion

The current investigation is the first to examine the developmental time course of body map formation in somatosensory cortex by recording from multiple sites in medial S1 in the same animal and relating these data to architectonic distinctions. We found that while cytochrome oxidase staining is useful for determining the medial to lateral anatomical parcellation within S1 at all stages between P5 to adults, other histological preparations provide similar results, although they work optimally at different developmental stages. Serotonin stains are most effective between P5 and P10, myelin basic protein stains provide the best results between P10 and P20, and myelin stains work well from P20 to adulthood. We also found that different structures within the brain scale linearly across development. For example, the relative size of the cerebral hemispheres, and the size of the cortical areas within a hemisphere, remains consistent throughout development compared to the entire brain or the entire cortical sheet, respectively. The final and most important observation of this study is that topographic maps of the body are not present at P5, in that most of the medial portion of S1 that would normally represent the hindlimb, forelimb and trunk in adults is dominated by the representation of the vibrissae. The emergence of topographic maps occurs over the subsequent two weeks and becomes adult like by P20. This observation is in contrast to the development of architectonic zones that normally correspond directly with different body part representations in the adult. These zones are present at P5, but clearly do not match the functional map of S1.

In the following discussion we first describe the gross morphological relationships between brain parts across development. We then discuss how anatomical parcellations develop in rat somatosensory cortex, and how our results on functional map development compares with other studies. Finally we discuss the broader issue of how topographic maps emerge and how the relationship between these maps and architectonic distinctions is established.

### Body, brain and cortical field size

We made comparisons of the whole brain, major structures of the brain, and cortical fields across cortical development and in adults. While all major portions of the brain, including the entire cortical hemisphere, the DLCS, and S1 as measured architectonically, became absolutely larger with age, the relative size of S1 as a function of the entire DLCS remained constant. Thus, major architectonically defined regions of the neocortex, such as S1, do not change their relative size across development.

### The development of anatomical parcellations in S1

In this discussion, it is important to make the distinction between anatomical parcellations within S1 and functional maps of S1. Anatomical or architectonic parcellations refer to the distinct regions within S1 that can be clearly defined based on different histological stains including Acetylcholinesterase (AChE), serotonin, CO, myelin, and Nissl to name a few. These regions have clearly demarcated boundaries that segregate them from other areas of the cortex and other subregions within S1. The term “functional maps” refers to the representation of the (most often) contralateral body in S1 that is defined using electrophysiological recording techniques. In adult animals, anatomical parcellations of S1 are coextensive with distinct functionally defined maps of the body, and these functional maps are topographically ordered [56], [57].

There are a number of studies that examine the development of anatomically defined cortical parcellations or fields and the molecular mechanisms that underlie cortical field emergence [58]. These studies demonstrate that as early as embryonic day (E) 21 AChE stains begin to reveal the anatomically defined barrels within S1. By P4 V1, A1 and S1 can be readily defined using serotonin and CO stains [59]–[61], and succinic dehydrogenase staining can be used to identify barrels by P5 [62]. Nissl stains only begin to reveal architectonic boundaries of fields by P12 [63]. Further, within S1, the anatomical zones that in adults are co-extensive with separate, functionally defined body part representations are clearly visible, and often viewed as intrinsically linked to the functional map of the body. The present results are in good agreement with these previous studies that examine the development of architectonic parcellations within S1 and extend them by demonstrating that in addition to CO, 5-HT staining can clearly identify V1, A1, and S1 at P5 and P10, and myelin basic protein works well to define barrels, although not other body parts at P10 and 15, and that adult like patterns of myelin are present by P20.

The contribution to different features of these anatomical parcellations from both sensory driven activity as well as genes intrinsic to the neocortex has been well studied for the barrel system in rodents [64], [65]. Early cortical patterning centers such as fibroblast growth factors (FGF), bone morphogenetic proteins (BMP) and the orthologs of vertebrate wingless (WNTs) define the major anterior/posterior and mediolateral axis of the cortex [11], [58], [66]–[69]. Secretion of molecules from these centers directs the graded expression of regulatory genes such as *GAP-43*, *Emx2*, *Pax6* and *Tbr1* [64], [70]–[72], which in turn regulate the region specific expression of other genes that encode cell adhesion molecules (cadherins) and axon guidance molecules (ephrins) [67], [73]–[75]. Important for this discussion, studies in which morphogens from early patterning centers, transcription factors, or downstream molecules have been manipulated either by deletion or over-expression demonstrate that the architectonic parcellations within S1 can be dramatically altered, and in the case of FGFs, translocated [68], [76]. In mice and rats most of these early genetic events occur embryonically or in the first postnatal days of life, well before thalamocortical axons have innervated layer 4 and sensory driven activity is fully present. Thus, the presence and general axis of anatomical parcellations of barrel cortex and cortex that will ultimately become the limbs and trunk is intrinsically mediated. But at this early stage, it is not directly related to functional maps of the body. The current study demonstrates that the association between anatomical parcellations and functional maps of the body is not complete until later postnatal development (between P15 and P20, see below).

In adult rats and mice, the barrel cortex is a first-order transformation of the whiskers on the contralateral face. Thus, dorsoventral rows of whiskers on the face are represented point-to-point as lateral to medial rows in cortex, and each barrel contains neurons with receptive fields on a single whisker [77]. When alterations to the whiskers are made early in development, such as the loss or over stimulation of a whisker, subsequent alterations in the anatomically defined barrels are observed. When whiskers are removed early in development, the barrel field will develop without the functional or anatomical representations of those whiskers [78]–[80]. Likewise, supernumerary whiskers, introduced through selective breeding, will be represented within the barrel field both functionally and anatomically [3], [12], [81]. Interestingly, removing all sensory input from the whiskers by infraorbital nerve cuts early in development (P0) results in a grossly disorganized barrel field in the cortex, but not the absence of a barrel field [82]. Given that the anatomical parcellections of barrels are present well before thalamocortical axons innervate layer 4, this is not surprising. This suggests that while sensory driven activity is necessary for its normal organization, and likely for the strict alignment of anatomically defined PMBS and functional maps of the whiskers, it is not necessary for its presence.

### Development of functional maps of the body

The studies that closely examine the relationship between whiskers on the face and anatomical segregates in the cortex form the foundation of our understanding of cortical map formation, and topographic ordering within the map. Despite this, there have been very few studies that have explicitly examined the development of whisker receptive fields for neurons within the barrels of the cortex [14], [15], [55], [83]. Further, we are aware of no studies that have tightly correlated the developmental time-course of the formation of anatomical parcellations with the emergence of full maps of the whiskers.

While this whisker/barrel system has offered important insights into cortical map development, synaptic development, and molecular and genetic mechanisms involved in arealization of the cortex, it is important to keep in mind that this is a highly derived system of murine rodents. Comparative studies demonstrate that this somatosensory specialization is present only in some, but not all rodents, and is not directly associated with whisking [7], [39]–[41]. Further, the presence of a barrel-like organization has only been observed in a few non-rodent mammals, such as the brush-tailed opossum [7], [84] and rabbits [7] and is thus independently evolved and homoplaseous rather than homologous. Taken together, these data suggest that the barrel field/whisker relationship is unique to a small selection of species and its organization and other characteristics may not be applicable to all mammals.

Surprisingly, we could find only two studies that examined the development of receptive field location and configuration for parts of the body other than the whiskers, and these were limited in scope. In 1975, Armstrong-James recorded from single cells in S1 of P7 rats [14]. He identified large receptive fields on multiple body parts, and determined that these receptive fields changed their size, shape, and orientation as he recorded from different cortical layers. Furthermore, he generated a composite map suggesting that S1 exhibited a rough topographic organization as early as P7. McCandlish et al. examined the age at which neurons within S1 first responded to somatosensory and/or electrical stimulation [15]. They determined that the development of neuronal responsiveness followed a lateral to medial gradient, with neurons responding to vibrissae or lip stimulation as early as 12 hours after birth, stimulation of the forelimb by 24 hours after birth, and stimulation of the hindlimb by 36 hours after birth. Further, while they did compare their functional maps to anatomical maps [15], [85], they did not directly compare functional maps to architectonic boundaries in the same animal in the same study.

While these studies were influential, they also had several shortcomings. First, both studies only focused on a single or limited number of developmental time points. Second, neither study generated dense electrophysiological maps in a single animal; they relied on composite maps made from the data gathered from multiple animals. Third, neither study correlated their functional composite maps with histologically processed tissue. While some aspects of our study are similar to these previous studies, other aspects are not; specifically, the size of receptive fields described by McCandlish and colleagues [15] for very early postnatal animals, (P0–P2) are very small and resemble receptive fields described for adult rats. This is puzzling because at this early stage, thalamocortical axons are dispersed in the subplate and cortical layer 4 has yet to form and become innervated [31].

The current study expands upon these previous studies by generating dense electrophysiological maps at multiple behaviorally significant developmental stages and relating those functional maps to anatomical parcellations. For the first time, we have demonstrated a dissociation between the development of functional and architectonic maps in the primary somatosensory cortex of rats. However, it should be noted that even while S1 was functionally disorganized, the vast majority of neurons that responded to somatosensory stimuli were found within the anatomical boundaries of S1. We observed some variability in the functional maps of S1 in early ages. This could be due to differences between litters, including but not limited to litter size, amount of maternal care, time of birth, and rate of maturation. It is not entirely surprising to see such variability at the youngest ages in our study. Since this is a period of extremely rapid growth and development, even seemingly small variations in rearing can have large consequences.

As noted above, most studies use the barrel cortex as a model for aspects of development, and specific to this portion of the discussion, thalamocortical development. While there are no studies that have examined thalamocortical development of medial S1 cortex, which normally represents the body, studies of thalamocortical development of barrels are useful in interpreting our findings on the over-representation of the whiskers in early postnatal animals. Thalamocortical axons begin to grow towards the developing cortex on E14 and have reached the cortical plate by E16-19 [86]–[88]. At the time they reach the cortical plate, layers 4 through 2/3 have yet to develop; this occurs postnatally at P4 [28], [89]–[91]. Using DiI placed in VPm, studies that examined patterns of thalamocortical afferentation of the cortex in early postnatal rats found that at P1, terminations form rows within what will become layer 4 of the barrel cortex, although no individual barrel pattern is present [87], [92]. By P4, thalamocortical axons terminate in a clear barrel like pattern in layer 4 [87]. Important for our discussion, there is clear evidence that at P4, VPm afferents do not terminate solely in the barrel field, but also in medial portions of S1, in what would be the hindlimb, trunk and forelimb representations in adult rats (see Figs. 3B and 1A in [92], [93], respectively).

These studies demonstrate that the anatomical substrate for an over-representation of the vibrissae in young postnatal animals is present. Unfortunately, there are no studies in rats that examine the development of thalamocortical afferents from VPl to the medial portions of S1. Studies that examine the development of connections from VPm and VPl in the same animal would be particularly useful, and would provide some insight into how and when these connections and the maps they generate become more restricted.

While the anatomical organization of S1 begins to emerge embryonically and is set by P4 [59]–[61], [87], as we have described here, the development of the functional organization of S1 lags significantly behind. Receptive fields for neurons within S1 become progressively smaller with age, and the functional map become co-extensive with the anatomical parcellations within S1. During the ages when the topographic maps are beginning to become adult-like (i.e., P10–20), the cortex is undergoing a series of dramatic changes. As described in the introduction, during this time GABA begins to have inhibitory effects [36]–[38], and inhibitory interneurons begin to play a role in cortical network activity [94]. The emergence of cortical inhibition may serve to restrict and redirect some of the “inappropriately” targeted neurons projecting throughout S1, resulting in refinement of S1 topography.

The exuberant growth of synapses that occurs during the second postnatal week [95], [96] is countered during the third postnatal week by the pruning of excess or underutilized connections. This process appears to be a general feature of neural development, and has been identified in muscles, autonomic ganglia, and the central nervous system [97]. Furthermore, synapse elimination through the Hebbian retraction of axonal collaterals is also occurring during this time [98]. Additionally, during this period synapses are more dynamic due to heightened LTP and LTD [33], [80]. It is probable that both pruning and synapse elimination also contribute to the refinement of the functional organization of S1.

Finally, it has been demonstrated that the functional organization of the barrel cortex goes through an experience-dependent critical period at the end of the second postnatal week, which may be influenced by increased dendritic spine plasticity [30], [34], [99]. It should be noted that this period of plasticity coincides with the onset of locomotor behavior and increased exploratory activity. As infant rats become more adept in using their bodies, some thalamocortical and corticocortical connections become strengthened through Hebbian processes, while other connections become weakened. This reorganization is potentiated by the increased plasticity that occurs during the critical period at the end of the second postnatal week. Thus, it is likely that the emergence of cortical inhibition, the pruning of exuberant connections, the elimination of unused synapses, increased LTP and LTD, and increased dendritic spine plasticity all contribute to the refinement of topographic maps that occurs between P10 and P20.

In conclusion, the formation of somatotopic maps within S1 appears to undergo three distinct developmental phases: genetically determined arealization of the cortex, activity dependent arealization of the cortex, and activity dependent refinement of the functional map. The findings of the current study support these contentions; the anatomical borders of S1 are clearly present at P5 while the maps of the body only become adult-like by the end of the third postnatal week. Thus, functional and architectonic maps, although seemingly inextricably linked during adulthood, in fact have very different developmental timelines and are dissociated during early development. It is clear that the criteria used to define receptive fields during adulthood (i.e., the convergence of architectonic and functional borders, and similarities in connections) cannot be used to define receptive fields in developing animals. This finding could have major implications for how cortical fields are defined during development.

## Materials and Methods

In these experiments 29 rats (*Rattus norvegicus*) were used to explore the development of topographic organization of the body representation within the primary somatosensory area (Table 2). Multi-unit electrophysiological recordings were combined with histologically defined boundaries so that the total extent of S1 and any topographic changes that occur throughout development could be quantified. All experiments were performed under National Institutes of Health guidelines for the care of animals in research and were approved by the Institutional Animal Care and Use Committee of the University of California, Davis (protocol #13263).

### Subjects

A total of 29 rats from 12 litters were used: 7 P5 rats (12.1–15.5 g), 6 P10 rats (24.0–28.5 g), 7 P15 rats (33.2–43.9 g), 5 P20 rats (45.2–66.0 g), and 4 adult rats (>P60; 275–360 g). All experiments were performed within 24 hours of subjects' reaching a given age. The sex and weights for all subjects are in Table 2. Data from all subjects were used for examining brain weights and brain/body weight ratios (i.e., neuroanatomical experiments), and data from a subset of subjects were used in both electrophysiological and neuroanatomical experiments (Table 2). Rats were housed in the vivarium at the University of California, Davis. Pups were born to Long Evans rats purchased from Harlan or Charles River, and were raised in litters that were culled to eight pups within three days of birth (day of birth was day 0; adult rats were >P60). Litters and pups were raised in standard laboratory cages in which food and water were available *ad libitum*. All rats were maintained on a 12-h light-dark cycle with lights on at 07.00 h.

### Electrophysiological Recordings

A total of 18 rats from 10 litters were used in these experiments; three each of P5 and P20 rats, and 4 each of P10, P15, and adult rats. A total of 958 recording sites were obtained for these animals with an average of 53.2 recordings per animal (Table 5). All animals were anesthetized with a dose of 30% urethane dissolved in propylene glycol (1.5 mg/kg IP) and/or isoflurane (1–3% in oxygen). Dexamethasone (0.4 mg/kg IM) was administered following anesthesia induction. Subcutaneous injections of lactated Ringer's solution (10 ml/kg/hour) were administered to maintain hydration. Body temperature was maintained and respiratory rate was monitored continuously throughout the experiment.

In P5 and P10 rats, after a surgical plane of anesthesia had been achieved, a craniotomy was performed exposing the left primary somatosensory cortex and the dura was removed. A custom-made device, which allowed the subjects' head to be securely fixed and stabilized, was attached to the skull using cyanoacrylate adhesive, and the device was secured to the stereotaxic frame. The exposed cortex was then coated with silicon fluid (Dow Corning 200 Fluid (dimethylpolysiloxane); Dow Corning, Midland, MI) and imaged with a digital camera (Nikon Coolpix 5700 or Nikon D300; Nikon, Inc., Melville, NY). This image was used as a reference map to relate the electrode penetrations to cortical vasculature.

In P15, P20, and adult rats, once anesthetized the subjects were placed in a stereotaxic apparatus. The skin was cut and the temporal muscle over the left hemisphere was retracted. A craniotomy was performed to expose S1 in the left hemisphere, and the dura was removed. The exposed cortex was coated with silicon fluid and imaged with a digital camera.

In all subjects a tungsten electrode (5 MΩ, 0.01 inch diameter, ALA scientific) was lowered into cortical layer 4. As the depth of layer 4 varied by age, the appropriate electrode depth was determined by response properties and previous examination of archival tissue from each age group. Multi-unit recordings were amplified (strongest recording ×5000) and filtered (100–5000 Hz; A-M Systems Model 1800 Microelectrode AC Amplifier; A-M Systems, Carlsborg, WA), heard through a speaker, and, in some cases, visualized and recorded using Spike2 (CED, Cambridge, UK). At each recording site responses to somatosensory stimulation were identified. Somatosensory stimulation consisted of light taps, displacement of hairs, light brushing of skin, hard taps, and manipulation of muscles and joints. Descriptions of the receptive fields and the type of stimulus required to elicit a response were documented and drawn on illustrations of the rat body. Each age group had its own set of illustrations. Responses were recorded at multiple, densely spaced recording sites (∼200–300 µm apart). The location of each recording site was marked on the digital image of the cortical surface relative to the vascular pattern, and was used to aid in the process of tissue reconstruction and receptive field quantification.

Upon completion of electrophysiological recordings, fluorescent probes (Fluororuby and/or Fluoroemerald, 7% concentration; Molecular Probes, Eugene OR) were placed at strategic locations in the cortex, and the placement of each probe was marked on the digital image of the cortex to aid with reconstruction of the tissue. Each recording session lasted for 2–4 hours.

### Histological Processing and Data Analysis

At the end of each recording session, the subject was euthanized with an overdose of sodium pentobarbital (250 mg/kg, IP) and perfused transcardially with 0.9% saline, followed by 4% paraformaldehyde in phosphate buffer (pH 7.4) and 4% paraformaldehyde in 10% sucrose in phosphate buffer. After fixation, the brain was extracted from the skull. The weight and volume of the whole brain, the left and right hemispheres (including the cortical sheet, hippocampus, and basal ganglia), and the subcortical regions (including the thalamus, hypothalamus, brainstem, and cerebellum) were taken. The two hemispheres were then flattened between two glass slides, and the flattened cortices were immersed in 30% sucrose overnight.

The flattened cortex was sectioned at 20–30 µm thickness in a plane parallel to the cortical surface. In all cases sections were stained for cytochrome oxidase (CO) [100]. Additionally, a myelin stain [101] as well as immunohistochemical serotonin (5-HT) and myelin basic protein stains (see methods below) were differentially performed on tissue from different aged rats.

### Immunohistochemical protocols

The 20–30 µm thick free-floating sections were first rinsed (3×5 min) in phosphate buffered saline (PBS). To quench endogenous peroxidase, sections were incubated in an aqueous solution of 10% MeOH and 3% H_2_O_2_ for 30 minutes at room temperature. Following rinses in PBS-0.1% Triton X-100 (3×10 min), non-specific binding was suppressed by a preincubation in 10% normal goat serum (NGS; Invitrogen, Camarillo, CA) and PBS-0.1% Triton X-100 for 1 hour at room temperature. Sections were then transferred to the primary antibody solution (Serotonin rabbit antibody, 1∶50,000; ImmunoStar, Hudson, WI; Rabbit polyclonal to Myelin Basic Protein, 1∶500; Abcam, Cambridge, MA) containing 10% NGS and PBS-0.1% Triton X-100 overnight at 4°C. Tissue sections were then rinsed in PBS-0.1% Triton X-100 (4×10 min) and incubated in the secondary antibody solution (Peroxidase-conjugated AffiniPure Goat Anti-Rabbit IgG, 1∶500; Jackson ImmunoResearch Laboratories, West Grove, PA) for 4 hours at room temperature. The tissue sections were thoroughly rinsed in PBS (3×10 min) and the secondary antibody binding was visualized using a standard DAB and hydrogen peroxide reaction. Sections were rinsed in PBS (3×5 min), mounted on gelatin-subbed slides, and coverslipped.

### Relating Electrophysiological Maps and Myeloarchitecture

In each case, camera lucida reconstructions of individual CO, 5-HT, and Myelin sections were made with a stereomicroscope (Zeiss Stemi SV6; Carl Zeiss Microimaging, Inc., Thornwood, NY). As described previously [51], whereas individual sections can contain many partial anatomical boundaries, the entire series of sections was examined and combined into a single comprehensive reconstruction to determine the full extent of cortical field boundaries (Fig. 4). Each reconstruction contained the outline of the section, blood vessels, tissue artifacts, probes, visible electrode tracks, and architectonic borders. Sections were aligned using these landmarks and compiled into one composite image. Architectonic boundaries and electrophysiological recordings were combined by aligning probes marked on the photograph of the brain with those visible in sectioned tissue to produce a comprehensive reconstruction.

The topographic organization of the primary somatosensory area was determined by examining the receptive fields for neurons at each electrode penetration and then grouping them by body part (i.e., head/vibrissae, forelimb, hindlimb, and trunk). Topographic maps of S1 were generated by correlating electrode penetrations with receptive field progressions for neurons.

### Data Analysis

In all subjects, the weights and volumes of the whole brain, left and right hemispheres, and subcortical regions were determined (Table 4) [102]. Developmental changes in the weights of the whole brain, left and right hemispheres, and subcortical regions, as well as the percentage of the whole brain comprised by the left and right hemispheres and subcortical regions were assessed using an analysis of variance (ANOVA; Excel; Microsoft, Redmond, WA), and differences between specific age groups were determined using unpaired *t*-tests. For all tests, alpha = 0.05.

In subjects that underwent electrophysiological mapping, the areas of different portions of the cortical hemispheres were measured, including the entire cortical sheet (which comprised the DLCS, pyriform cortex, and the olfactory bulb; Table 3), the DLCS, and S1, as determined by its architectonic boundaries (ImageJ; NIH, Bethesda, MD). The percentage of the DLCS occupied by S1 (S1%) was also calculated. Developmental changes in the size of the entire cortical sheet, DLCS, S1, and the percentage of DLCS occupied by S1 were analyzed for all age groups using ANOVA, and differences between specific age groups were determined using unpaired *t*-tests. For all tests, alpha = 0.05.
